# The congenital hearing phenotype in *GJB2* in Queensland, Australia: V37I and mild hearing loss predominates

**DOI:** 10.1038/s41431-024-01584-0

**Published:** 2024-03-15

**Authors:** Rebecca Kriukelis, Michael T. Gabbett, Rachael Beswick, Aideen M. McInerney-Leo, Carlie Driscoll, Karen Liddle

**Affiliations:** 1 Queensland Children’s Hospital, South Brisbane, QLD Australia; 2 Centre for Genomics and Personalised Health, School of Biomedical Sciences, Queensland University of Technology, Brisbane, QLD Australia; 3 University of Queensland Centre for Children’s Health Research, South Brisbane, QLD Australia; 4 Healthy Hearing Program, Children’s Health Queensland Hospital and Health Service, Brisbane, QLD Australia; 5 School of Health and Rehabilitation Sciences, University of Queensland, Brisbane, QLD Australia; 6 Frazer Institute, University of Queensland, Dermatology Research Centre, Brisbane, QLD Australia

**Keywords:** Genetics research, Paediatrics, Genetic testing, Genetic counselling

## Abstract

*GJB2* was originally identified in severe, non-syndromic sensorineural hearing loss (SNHL), but was subsequently associated with mild and moderate SNHL. Given the increasing utilisation of genetic testing pre-conceptually, prenatally, and neonatally, it is crucial to understand genotype-phenotype correlations. This study evaluated the nature and frequency of *GJB*2 variants in an Australian paediatric population with varying degrees of SNHL ascertained through newborn hearing screening. Audiograms from individuals with *GJB2* variants and/or a *GJB6* deletion (*GJB6*-D13S11830) were retrospectively reviewed (*n* = 127). Two-thirds were biallelic (homozygous/compound heterozygous) for pathogenic/likely pathogenic variants of *GJB2* and/or *GJB6* (*n* = 80). The most frequent variant was c.109 G > A, followed by c.35delG and c.101 T > C. Compared to biallelic carriage of other *GJB2* variants, c.109 G > A positive individuals (homozygous/compound heterozygous) were more likely to have mild HL at their initial and latest audiograms (*p* = 0.0004). Biallelic carriage of c.35delG was associated with moderately-severe or greater SNHL at both initial and latest audiograms (*p* = 0.007). The c.101 T > C variant presented with milder SNHL and U-shaped audiograms (*p* = 0.02). In this agnostically identified cohort, mild SNHL predominated in *GJB2/GJB6* carriers in contrast to previous studies targeting individuals with significant loss. Consequently, c.109 G > A, associated with milder phenotypes, was the most frequent. This study provides valuable data to support prognostic confidence in genetic counselling.

## Introduction

Sensorineural hearing loss (SNHL) is the most common congenital sensory disorder, affecting 1 in 500 newborns [1]. It can adversely affect language development, cognition, psychosocial wellbeing and quality of life, educational attainment, and economic independence at various stages of life [2]. Approximately 50% of congenital hearing loss is Mendelian, with 30% of individuals having a recognised syndrome and 70% regarded as non-syndromic [3].

Non-syndromic hearing loss (NSHL) is heterogeneous with approximately 75% of early onset cases being inherited in an autosomal recessive manner [4]. Seventy-eight of the 124 NSHL genes are autosomal recessive [5], and the most frequently implicated gene is *GJB2* (OMIM 121011), which is responsible for the protein gap junction protein beta-2 (connexin 26). However, a single *GJB2* variant can lead to disease if coinherited with a variant in the contiguous gene *GJB6*, (OMIM 604418) gap junction protein beta-6 (connexin 30) in approximately 2% of cases [5, 6]. In addition, pathogenic variants in *GJB2* can be associated with autosomal dominant inheritance in approximately 2% of cases [7].

Worldwide, *GJB2* pathogenic variants account for 18–50% of prelingual NSHL [8]. In addition, *GJB2* variants account for 30-50% of all cases of profound NSHL [9]. Widespread testing of *GJB2* (and *GJB6*), reveals variable degrees of hearing loss (mild to profound), not always detectable at birth, which is usually bilateral, but occasionally unilateral, and can be progressive [3, 8].

Previous cohort studies identified a recurrent variant in *GJB2* (c.35delG) estimated to account for approximately 60-70% of deafness in European, North African, Middle Eastern, Asian, North and South American populations [8, 10, 11]. A single study in an Australian paediatric population identified the c.35delG variant to be the most frequently implicated (38.03%) [12]. Other *GJB2* variants have been implicated in other populations. Specifically, the c.235delC and c.109 G > A *GJB2* variants are more frequently implicated in hearing loss in East Asian countries including Japan, Korea and China [10].

Mode of ascertainment affects the phenotypic spectrum associated with specific genes, where they are originally described in the most severe cases and, through wider testing practices are subsequently associated with more variable phenotypes, thus broadening the phenotypic spectrum [13, 14]. In some cases, variants are identified in healthy individuals who might never develop symptoms [13]. Thus, context (both timing of testing and target population) will affect genotypic and phenotypic findings, and the clinical significance of the results. This is highly relevant to the *GJB2* story which was originally identified with predominantly severe/profound HL [15]. Nowadays, we are using genetic testing generally and *GJB2* specifically in population screening. Many countries, including Australia, now have mature newborn hearing screening programs with high population coverage that result in identification of infants with mild congenital HL [16]. In addition, reproductive carrier screening has become more affordable, and many commercially available panels include *GJB2* [17]. Therefore, it is vitally important to publish results from a range of contexts to ensure that clinicians have access to data from individuals with milder or minimally apparent phenotypes to ensure prognostic confidence in the likely spectrum of outcomes for the individuals undergoing screening [18].

There is significant genotypic and phenotypic variability in international studies, but limited data from Oceania. Of note, a recent systematic review identified that only 0.7% of publications about connexin gene variants were from Australia [11]. Thus, this study reviewed all *GJB2* positive cases from the Queensland state laboratory, documented all *GJB2* and *GJB6* genotypes, and reviewed audiological and clinical data to identify possible genotype-phenotype correlations for a Queensland paediatric population with NSHL.

## Methods

### Study population and context

Queensland is an Australian state, where 99% of the 60 000 babies born each year, have newborn hearing screening as part of a state-government funded program (‘Healthy Hearing Program’). Screen positive individuals are referred for follow up diagnostic assessment in accordance with a state-wide protocol https://www.childrens.health.qld.gov.au/resources/our-work/healthy-hearing/queensland-health-screening-protocols-and-guidelines [19]. This study includes children diagnosed with a HL through this pathway or those diagnosed with SNHL in later childhood (either through the targeted surveillance program or external referral). Included individuals were seen in specialised paediatric ENT and/or medical hearing loss clinics at Queensland Children’s Hospital and other public hospitals statewide who had subsequent genetic testing with Pathology Queensland. All individuals had undergone genetic testing between November 2014 and December 2019. Medical and audiological records of this paediatric population were retrospectively reviewed. Ancestral background information was not routinely available; however, recent census data shows that whilst English and Australian ancestry is more common (33.6% and 31.2% respectively), approximately 17.4% of the population identify as Asian with breakdowns of 6.5% from Southern and Central Asia, 6.4% from North-East Asia and 4.5% from South-East Asia. Of note, First Nations peoples (Aboriginal and Torres Strait Islander peoples) were recorded at 3.2% of 2021 census [20].

### Genetic testing

Pathology Queensland is a state-wide service offering genetic testing for individuals diagnosed with HL. Specifically, the entire coding region of *GJB2* is sequenced (Sanger sequencing) for all patients and no further testing is conducted on individuals found to carry biallelic variants. When *GJB2* heterozygosity is identified, there is subsequent screening for a single *GJB6* deletion (del(*GJB6*-D13S11830) due to the interactive association. The study was confined to all patients that undertook *GJB2* and *GJB6* testing during the study period. Patients with no detected variants were excluded from the study.

Medical records were reviewed for all cases including homozygous/compound heterozygous and heterozygous cases or a single *GJB2* variant in the presence of a *GJB6* deletion. Variant pathogenicity was initially evaluated by reviewing ClinVar [21] and the Deafness Variation Database [22] to determine prior association with disease. For all variants not reported in ClinVar, a Varsome assessment [23] rated their likely pathogenicity using the American College of Medical Genetics and Genomics guidelines and best practices for expert interpretation of genomic data [24]. Based on these categories individuals were classified as autosomal dominant pathogenic variant, homozygous for pathogenic variants, compound heterozygous for two pathogenic variants, compound heterozygous for two variants (at least one pathogenic), digenic (one *GJB2*, one *GJB6*), heterozygous for pathogenic/likely pathogenic variants or heterozygous for VUS/likely benign/benign variant. The hearing profiles were included for all variant carriers, but statistical analyses (see below) were limited to individuals with biallelic pathogenic/likely pathogenic variants.

### Hearing assessment

Information relating to the severity of HL and a description of the audiogram was collated. The sensorineural (permanent) component of the HL was used in the cases with mixed (both sensorineural and conductive) HL, and we aimed to exclude temporary conductive HL, but the distinction was not always apparent during early testing. Degrees of HL were based on the classification system outlined by Goodman and Clark and include normal (≤20 dB HL), mild (21–40 dB HL), moderate (41–55 dB HL), moderately severe (56–70 dB HL), severe (71–90 dB HL), and profound (>90 dB HL). A four-frequency average was used to determine the degree of hearing loss [25, 26]. The audiograms were also assessed for shape (rising, sloping/descending, flat, U-shaped), symmetry (symmetrical, asymmetrical) and stability (fluctuating, progressive, stable) [27, 28]. (See Supplementary Table 1 for definitions of descriptors). If the HL was asymmetrical, it was graded according to the better hearing side. Audiometric testing was performed using a variety of age-appropriate standardised techniques for paediatric populations https://www.childrens.health.qld.gov.au/resources/our-work/healthy-hearing/audiology-diagnostic-assessment-protocol [19]. In cases where consecutive reports were available, the stability of the HL was also documented, by comparing audiogram results from the initial and most recent hearing assessment. Only patients with copies of audiograms in their medical record were included in the study. Audiology data was confirmed and supplemented through review of the QChild database, Queensland’s newborn hearing screening data management system which contains demographic and clinical information from screening.

### Data analysis

All genetic and phenotypic data (extracted from patient medical records and QChild) were exported into Microsoft Excel. A descriptive statistical analysis was performed. Fisher exact statistical tests were used to determine whether specific genotypes were more frequently associated with milder or more severe audiological phenotypes as well as other audiological descriptors. Specific genotype-phenotype analysis was performed for individuals who had pathogenic or likely pathogenic variants that were inherited in a homozygous, compound heterozygous or autosomal dominant manner. Moderately-severe, severe and profound HL were grouped together due to sample size limitations. A *p* value < 0.05 was considered statistically significant.

## Results

### Study population

Of the 625 individuals with *GJB2*+/− *GJB6* sequencing through Pathology Queensland during the study period (2014 to 2019), 134 had variants detected and sufficient audiological information. After review of patient medical records and QChild, seven patients had other known identifiable causes for their HL (three with absent cochlear nerves, two with enlarged vestibular aqueducts, one with Waardenburg syndrome and another with a chromosome 15q11.1 deletion) and were excluded. This left a total of 127 patients for analysis.

### Demographics

The final 127 cohort consisted of 72 males and 55 females. The mean age at genetic testing was 1.8 years with the range between 2 months and 17 years. The median age of genetic testing was 4 months with 68% of the cohort having the testing under 12 months of age. Specific information about ancestral background was not consistently available.

### Genetic Findings

In the cohort of 127 patients, 36 different *GJB2* variants were identified in a homozygous (*n* = 45/127, 35.4%), compound heterozygous (*n* = 36/127, 28.3%), 1 digenic (heterozygous for *GJB2* combined with the *GJB6* del(*GJB6*-D13S11830) deletion) and autosomal dominant (*n* = 1) or heterozygous (*n* = 44/127, 34.6%) state. Of the 36 individuals with compound heterozygous variants, 3 had only one of the variants rated as pathogenic. Of the 44 individuals with heterozygous variants, 22 had variants that were pathogenic or likely pathogenic and 22 had variants that were of unknown significance, benign or likely benign. These 47 individuals were included in the demographic analysis but were treated separately in the genotype-phenotype analysis.

In total, only 80 individuals were biallelic for pathogenic/likely pathogenic variants including one heterozygous with autosomal dominant inheritance.

**Fig. 1:**
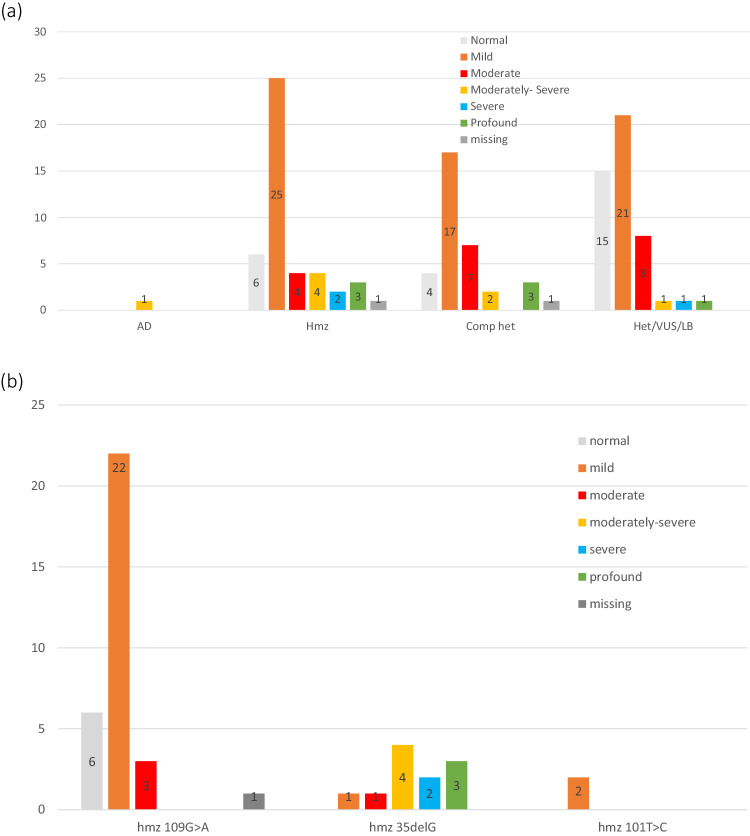
Genotype Phenotype: Initial HL by zygosity. **a** Whole cohort. **b** Homozygous group.

Three recurrent variants, c.109 G > A p(Val37Ile), c.35delG p.(Gly12Valfs*2), and c.101 T > C p.(Met34Thr) accounted for 48.4% (*n* = 77/159), 31.4% (*n* = 50/159) and 15.5% (*n* = 25/159) of all pathogenic or likely pathogenic variant alleles respectively. Furthermore, the c.109 G > A variant accounted for the majority of homozygous cases (*n* = 32/45, 71.1%). (See Fig. 1b). Table 1 demonstrates the distribution of the variants in the cohort for the 80 individuals with biallelic variants. Details of the remaining 47 individuals are presented in Supplementary Table 2.

**Table 1:** Genotypes and phenotypes with age at genetic testing for individuals with biallelic or heterozygous (autosomal dominant) variants in *GJB2* NM_004004.5 (NP_003995.2) *n* = 80.

Zygosity	Age at genetic testing (years)	Variant(s)	Initial HL degree (better ear)	Latest HL degree (better ear)	ACMG	ClinVar*
Autosomal Dominant	0 years, 5 months	c.551 G > A p.(Arg184Gln)	mod-severe	severe	P	P
Homozygous	0 years, 3 months	c.35delG p.(Gly12Valfs*2)	profound	profound	P	P
	0 years, 2 months		profound	profound		
	0 years, 3 months		mod	mod		
	0 years, 2 months		severe	missing		
	0 years, 2 months		mild	missing		
	0 years, 3 months		mod-severe	severe		
	0 years, 2 months		severe	severe		
	0 years, 6 months		mod-severe	mod-severe		
	0 years, 1 months		mod-severe	mod-severe		
	0 years, 2 months		profound	profound		
	0 years, 2 months		mod-severe	mod-severe		
	9 years, 4 months	c.109 G > A p.(Val37Ile)	mild	mild	P	P
	3 years, 8 months		mild	mild		
	7 years, 3 months		mild	mild		
	7 years, 2 months		mild	mild		
	6 years, 6 months		mild	mild		
	2 years, 8 months		normal	normal		
	2 years, 1 months		mild	missing		
	3 years, 11 months		mild	mild		
	2 years, 4 months		normal	mild		
	13 years, 9 months		mild	mild		
	0 years, 8 months		mod	mod-severe		
	0 years, 3 months		mild	mild		
	0 years, 1 months		mild	mild		
	0 years, 6 months		mild	mild		
	0 years, 2 months		mild	mild		
	2 years, 11 months		normal	normal		
	2 years, 6 months		missing	mild		
	0 years, 1 months		normal	normal		
	1 years, 4 months		normal	missing		
	0 years, 2 months		mod	mod		
	0 years, 4 months		mild	mild		
	0 years, 8 months		mild	mild		
	0 years, 2 months		mild	mild		
	0 years, 4 months		mild	mild		
	0 years, 2 months		mild	mild		
	1 years, 3 months		mild	mild		
	0 years, 5 months		mild	mild		
	0 years, 4 months		mild	mild		
	0 years, 5 months		mild	mild		
	0 years, 3 months		mod	mod		
	0 years, 3 months		normal	normal		
	0 years, 2 months		mild	mild		
	0 years, 4 months	c.101 T > C p.(Met34Thr)	mild	missing	P	P
	0 years, 3 months		mild	mild		
Compound heterozygous or Digenic (pathogenic variants)	0 years, 2 months	c.71 G > A p.(Trp24*) c.250 G > C p.(Val84Leu)	profound	profound	P P	P P
	0 years, 1 months	c.71 G > A p.(Trp24*) c.458_475dup p.(Val153_Tyr158dup)	profound	profound	P P	P P
	5 years, 8 months	c.101 T > C p.(Met34Thr) c.109 G > A p.(Val37Ile)	mild	mild	P P	P P
	0 years, 1 months	c.101 T > C p.(Met34Thr) c.298 C>Tp.(His100Tyr)	mod	mod	P P	P P
	4 years, 1 months	c.109 G > A p.(Val37Ile) c.508_511dup p.(Ala171Glufs*40)	normal	mild	P P	P P
	0 years, 3 months	c.235delC p.(Leu79Cysfs*3) c.299_300delAT p.(His100Argfs*14)	mod	mod	P P	P P
	0 years, 4 months	c.250 G > C p.(Val84Leu) c.-23 + 1 G > A	mod	mod	P P	P P
	0 years, 2 months	c.35delG p.(Gly12Valf*2) c.-23 + 1 G > A	mild	missing	P P	P P
	0 years, 6 months	c.35delG p.(Gly12Valf*2) c.101 T > C p.(Met34Thr)	normal	mild	P P	P P
	1 years, 4 months		mild	mild		
	0 years, 2 months		mod	mod		
	0 years, 2 months		mild	missing		
	6 years, 11 months		mild	mild		
	5 years, 6 months		mild	mild		
	1 years, 5 months		normal	mild		
	0 years, 2 months		mod	mod		
	0 years, 3 months		mild	missing		
	0 years, 2 months		mild	mild		
	3 years, 7 months		mild	missing		
	17 years, 8 months	c.35delG p.(Gly12Valfs*2) c.139 G > T p.(Glu47*)	mod-severe	missing	P P	P P
	0 years, 1 months		mod	severe		
	10 years, 10 months	c.35delG p.(Gly12Valfs*2) c.109 G > A p.(Val37Ile)	mod	mod	P P	P P
	0 years, 6 months		mild	mild		
	0 years, 7 months	c.35delG p.(Gly12Valfs*2) c.313_326del p.(Lys105Glyfs*5)	profound	missing	P P	P P
	0 years, 1 months	c.35delG p.(Gly12Valfs*2) c.269 T > C p.(Leu90Pro)	mild	missing	P P	P P
	4 years, 2 months		normal	mild		
	0 years, 1 months		mild	missing		
	0 years, 3 months		mild	mild		
	0 years, 3 months	c.35delG p.(Gly12Valfs*2) c.169 C > T p.(Gln57*)	mild	mild	P P	P P
	0 years, 3 months	c.35delG p.(Gly12Valf*2) c.235delC p.(Leu79Cysfs*3)	mod-severe	mod-severe	P P	P P
	0 years, 2 months	c.109 G > A p.(Val37Ile) c.583 A > G p.(Met195Val)	mild	normal	P LP	P
	0 years, 2 months	c. 34 G > T p.(Gly12Cys) c.109 G > A p.(Val37Ile)	mild	normal	LP P	LP P
	1 years, 1 months	c.101 T > C p.(Met34Thr) c.194 A > G p.(Tyr65Cys)	mild	normal	P LP	P LP
	0 years, 2 months	c.101 T > C p.(Met34Thr) del(GJB6-D13S11830)	mild	mild	P P	P P

*as at July 2023.

A single patient carried del(*GJB6*-D13S11830), in conjunction with a *GJB2* c.101 T > C variant. The autosomal dominant variant, c.551 G > A, was identified in a case whose mother also had SNHL and had been previously reported in association with DFNA3 [29].

### Biallelic cases: Hearing profiles

The specific genotype-phenotype analysis was performed on 80 individuals with biallelic or an autosomal dominant variant. The mean age at testing for this group was 1.8 years with a median age at testing of 4 months. The most frequent loss in the better ear was mild 43/80 (53.8%), with 10/80 initially coded as normal in the better hearing ear (12.5%), 11/80 moderate (13.8%), 7/80 moderately severe (8.8%), 2/80 severe (2.5%) and 6/80 profound (7.5%) (Fig. 1a). For the initial hearing assessments, one patient had missing data, but was included because their subsequent audiogram was available. 60/80 patients had subsequent audiogram data available to evaluate change in hearing over time. Proportions of children in the severity categories was similar between initial and latest assessments. (Supplementary Figure 1). Note that overall hearing was defined by the better hearing ear. 60 of the 80 patients in this group had information about stability and 49 of those had stable hearing profiles (49/60 = 81.6%) with most having mild HL (*n* = 25/60, 41.6%). Two patients had fluctuating hearing profiles with mild HL on their latest test (*n* = 2/60, 3.3%). Both individuals were homozygous for the c.109 G > A variant. The other 9 individuals (*n* = 9/60, 15%) had progressive HL. Further information can be found in Table 2a.

**Table 2:** a: Genotype-phenotype correlations for progressive hearing loss *n* = 15 (15/127 = 11.8% of the cohort), b: Genotype Phenotype correlations with initial hearing loss coded as ‘normal’ (*n* = 25 25/127 = 19.6% of the cohort).

Genotype	Zygosity	Number (%) of children	Degree of HL initial	Degree of HL latest
Homozygous and compound heterozygous (Pathogenic/Likely Pathogenic) n = 9				
c.551 G > A p.(Arg184Gln)	Autosomal dominant	1	moderately-severe	severe
c.109 G > A p(Val37Ile)	hmz	2	mild	mild (deterioration in low frequencies)
c.35delG p.(Gly12Valfs*2)	hmz	1	moderately-severe	severe
c.35delG p.(Gly12Valfs*2) c.101 T > C p.(Met34Thr)	comp het	2	2 normal	2 mild (U-shaped)
c.35delG p.(Gly12Valfs*2) c.139 G > T p.(Glu47Ter)	comp het	1	moderate	severe (cochlear implants)
c.35delG p.(Gly12Valfs*2) c.269 T > C p.(Leu90Pro)	comp het	1	normal	mild
c.109 G > A p(Val37Ile) c.508_511dup p.(Ala171Glufs*40)	comp het	1	normal	mild (U-shaped)
Heterozygous (pathogenic/likely pathogenic) n = 4				
c.35delGp.(Gly12Valfs*2)	het	1	mild	moderate
c.101 T > C p.(Met34Thr)	het	2	1 normal; 1 moderate	2 moderately-severe sloping
c.313_326del p.(Lys105Glyfs*5)	het	1	normal	mild
Heterozygous (likely benign/benign) n = 2				
c.-216T > G p.(=)	het	1	moderate	Moderately -severe
c.79 G > A p.(Val27Ile)	Het	1	normal	severe

Genotype	Zygosity	No of children	Degree of HL initial ‘normal’	Degree of HL latest
Homozygous and compound heterozygous (pathogenic/likely pathogenic) n = 10				
c.109 G > A p(Val37Ile)	hmz	6	1 normal, 3 slight, 2 unilateral	1 unilateral, 3 slight 1 mild 1 missing
c.109 G > A p(Val37Ile) c.508_511dup p.(Ala171Glufs*40)	comp het	1	unilateral	bilat mild to mod
35delG p.(Gly12Valfs*2) c.101 T > C p.(Met34Thr)	comp het	2	unilateral	Progressed to bilat U-shaped
35delG p.(Gly12Valfs*2) c.269 T > C p.(Leu90Pro)	comp het	1	normal	bilat mild rising
Compound heterozygous (one pathogenic variant) and heterozygous n = 7				
c.109 G > A p(Val37Ile) c.265 C>Tp.(Leu89Phe)	het	1	slight L	normal
c.109 G > A p(Val37Ile) c.571 T > C p.(Phe191Leu)	het	1	Unilateral L high frequency	L U-shaped, R rising
35delG p.(Gly12Valfs*2)	het	2	1 unilateral, 1 slight	1 unilateral, 1 missing
c.101 T > C p.(Met34Thr)	het	1	normal	moderately-severe bilateral
c.313_326del p.(Lys105Glyfs*5)	het	1	unilateral high frequency	bilateral mild
c.514 T > A p.(Trp172Arg)	het	1	unilateral	unilateral
Heterozygous (VUS/LB/B) n = 8				
c.79 G > A p.(Val27Ile)	het	5	1 normal, 2 unilateral, 1 slight, 1 temporary conductive	1 unilateral, 1 temporary conductive 1 bilateral profound 2 missing
c.341 A > G p.(Glu114Gly) c.79 G > A p.(Val27Ile)	het	1	unilateral	missing
c.-45C > A	het	1	unilateral	unilateral
c.-130C > G	het	1	temporary conductive	temporary conductive

*Hmz* homozygous, *comp het* compound heterozygous.

*Comp het* compound heterozygous, *VUS* variant of uncertain significance, *LB* likely benign, *B* benign.

Ten of 80 (12.5%) patients demonstrated hearing that was coded as ‘normal’ in the better hearing ear at their initial hearing test. The genotype and further information about HL in these individuals is presented in Table 2b. The majority of these individuals had progression of their HL.

### Heterozygous cases: Hearing profiles

The hearing profiles in heterozygous cases can be seen in Supplementary Table 2, Fig. 1a and Supplementary Figure 1b. Of the 47 individuals with heterozygous variants and those with variants of uncertain significance or likely benign, the hearing profiles were similar between the initial and latest assessment with nine not having follow up audiogram data. The proportions of SNHL severity at the initial time point were 15/47 (31.9%) normal in the better ear (more detail in Table 2) mild 21/47 (44.7%), moderate 8/47 (17%) and one each of moderately-severe (2.1%), severe (2.1%) and profound (2.1%) (Supplementary Figure 1b).

HL progression was assessable in 97 individuals (from both biallelic and heterozygous groups) who had audiograms at multiple time points and sufficient information to code HL stability. Progressive HL was seen in 15 total; 9/60 (15%) in the homozygous/compound heterozygous/AD group and 6/37 (16.2%) in the heterozygous/VUS/likely benign group. Genotypes of those individuals are presented in Table 2a and include the autosomal dominant variant, homozygous and compound heterozygous c.35delG and c.109 G > A as well as several other genotypes.

### Biallelic cases: genotype-phenotype associations

The three most common variants had sufficient sample size for Pearson’s chi-squared or Fisher’s exact statistical test analysis for association between genotype and phenotypic characteristics and were all present in homozygous, compound heterozygous and heterozygous states (More detail in Table 1). Figure 1a demonstrates the degree of initial HL by zygosity and 1b demonstrates the degree of initial HL for the three most frequent variants in homozygous state. The most frequent variant, c.109 G > A, had sufficient numbers for analysis for phenotypic associations with both homozygous state and combination of homozygous/compound heterozygous, and c.35delG and c.101 T > C had sufficient numbers for this analysis of homozygous/compound heterozygous state.

Fisher exact statistical test analysis found that patients who were homozygous for c.109 G > A were much more likely to have mild HL and less moderately severe/severe/profound for both their initial and latest hearing tests (*p* = 0.0004 and 0.006 respectively) than the rest of the cohort. Individuals who were homozygous/compound heterozygous for the c.109 G > A variant were also significantly more likely to have mild HL (versus moderately-severe/severe/profound HL) as compared to those not carrying the c.109 G > A variant for both the initial (*p* = 0.00004) and most recent audiogram data (*p* = 0.0004) (Table 3).

**Table 3:** Degree of HL initial and latest for patients (i) homozygous for *GJB2* c.109 G > A p.(Val37Ile), (ii) homozygous or compound heterozygous c.109 G > A p.(Val37Ile) and (iii) homozygous or compound heterozygous c.35delG p(Gly12Valfs*2) variant present (Fisher’s exact statistical test).

	c. 109 G > A hmz/comp het versus heterozygous or negative						c.35delG hmz/comp het versus heterozygous/negative cases		
Characteristic	Homozygous c.109 G > A variant (*N* = 32)	negative, comp het or het for c.109 G > A (*N* = 48)	p-value*	All Hmz and comp het c.109 G > A (*N* = 38)	Negative or het for c.109 G > A *N* = 42	p-value*	Hmz or comp het c.35delG (*N* = 34)	negative or het for c.35delG (*N* = 46)	*p*-value*
Degree of Hearing Loss- Initial			0.0004			0.00004			0.007
Normal	6 (19%)	4 (8.3%)		7 (18.9%)	3 (7.1%)		3 (9.1%)	7 (15.2%)	
Mild	22 (71%)	21 (43.8%)		26 (70.2%)	17 (40.5%)		13 (39.4%)	30 (65.2%)	
Moderate	3 (9.7%)	8 (16.7%)		4 (10.8%)	7 (16.7%)		5 (15.2%)	6 (13%)	
Moderately- Severe/Severe/Profound	0 (0%)	15 (31.3%)		0 (0%)	15 (35.7%)		12 (36.4%)	3 (6.5%)	
Unknown	1	0		1	0		1	0	
Degree of Hearing Loss- Latest			0.006			0.0004			0.007
Normal	4 (13.3%)	3 (8.6%)		6 (16.7%)	1 (3.4%)		0 (%)	7 (16.7%)	
Mild	23 (76.7%)	15 (%)		26 (72.2%)	12 (41.4%)		11 (47.8%)	27 (64.3%)	
Moderate	2 (6.7%)	7 (20%)		3 (8.3%)	6 (20.7%)		4 (17.4%)	5 (11.9%)	
Moderately- Severe/Severe/Profound	1 (3.3%)	10 (28.6%)		1 (2.8%)	10 (34.5%)		8 (34.8%)	3 (7.1%)	
Unknown	2	13		2	13		11	4	

*Fisher’s exact probability test.

Individuals who were homozygous/compound heterozygous for the c.35delG variant had significantly more moderately-severe/severe/profound HL than those with other genotypes at both time points (p = 0.007 and 0.007 respectively) (Table 3).

Individuals who were homozygous/compound heterozygous for the c.101 T > C variant had significantly more U-shaped and sloping audiograms than other audiogram configurations at the most recent time point (*p* = 0.02) (Table 4). There were no other significant associations between genotype and any of the other audiology descriptors (audiogram shape, symmetry or stability).

**Table 4:** Audiogram shape initial and latest for patients: c.101 T > C p(Met34Thr) homozygous/compound heterozygous variant present (Fisher’s exact statistical test).

Characteristic	Hmz/comp het c.101 T > C Present: *N* = 17	negative or het for c.101 T > C *N* = 63	*p*-value*
Audiogram shape- initial			0.14
Flat	7 (43.8%)	39 (61.9%)	
Rising	0 (0%)	5 (7.9%)	
Sloping	7 (43.8%)	17 (27%)	
U-shaped	2 (12.5%)	2 (3.2%)	
Unknown	1	0	
Audiogram shape- Latest			0.02
Flat	3 (21.4%)	26 (53.1%)	
Rising	0 (0%)	4 (8.2%)	
Sloping	6 (42.9%)	15 (30.6%)	
U-shaped	5 (35.7%)	4 (8.2%)	
Unknown	3	14	

*Fisher exact probability test.

### Genotype-Phenotype association: progressive HL and ‘normal’ hearing

Of the 10 individuals who were homozygous/compound heterozygous who had normal hearing in the better ear on the initial audiogram (Table 2b), 6 were homozygous for c.109 G > A. There were 2 individuals who were compound heterozygous for c.35delG/c.101 T > C who had unilateral HL at the initial time point and bilateral U-shaped HL at the most recent time point. Six of the 15 individuals with progressive HL (Table 2a) had at least one variant that was c.35delG.

## Discussion

This study reports variant data from a geographical region which has been understudied to date. State-wide newborn screening (99% coverage), and follow-up *GJB2/6* testing in screen positive individuals revealed biallelic and heterozygous carriage in association with predominantly mild hearing loss. The most frequent variant, c.109 G > A, has been described with mild phenotypes, and may partially explain the increase in mild hearing loss detected on newborn screening. Consistent with the literature, the c.35delG variant was associated with more severe HL, while the c.101 T > C variant was associated with milder HL and U-shaped audiograms. This information provides a more complete picture of the phenotypic spectrum of *GJB2/6* associated HL which provides short-term prognostic data and can inform pre- and post-natal counselling for individuals and families found to carry these variants.

Ascertainment in this study differs from most previously reported literature which genetically evaluated individuals being considered for cochlear implants i.e., typically severe levels of HL [30, 31]. In those studies, the most common variants were c.35delG and c.235delC which were identified in populations from European and Asian backgrounds, respectively. The c.109 G > A variant has been reported previously, especially from Asian ancestry cohorts [32], where the minor allele frequency is 0.08 [33]. Thus, it is unsurprising that it is prevalent in our agnostically ascertained cohort where the sensitive equipment used for universal newborn hearing screening and aABR (automated auditory evoked brainstem response) as a screening method can lead to capturing patients that may have mild, transient and/or fluctuating hearing profiles.

An artefact of newborn screening is the detection of mild hearing loss. As mild HL is being increasingly diagnosed at an earlier age [34], this presents prognostic and management uncertainty for both families [35] and clinicians [16]. This study provides evidence for a genetic basis for many mild HL cases adding to the emerging body of literature describing genotypes in mild and moderate HL cohorts [36, 37].

Prior publications have noted milder HL in association with either c.101 T > C or c.109 G > A alleles [38]. The pathogenic classifications of both variants were initially controversial, but an international consensus paper classified both as pathogenic with variable expressivity and incomplete penetrance [39]. Consistently, in this Australian cohort this variant is associated with a milder phenotype. This phenotypic information is valuable for clinicians and families presented with these results in infancy, prenatally or as part of reproductive carrier screening [40].

Previous research has shown that a heterozygous *GJB2* variant is detected in 10-50% of individuals with HL [41], which can complicate and limit clinical interpretation and management. Additionally, some studies have indicated that carriers of certain variants have been reported to be more likely than ‘non-carriers’ to develop HL when exposed to other environmental factors or genetic defects [42]. In the current study, heterozygosity was identified in 37% of the cohort and the associated HL phenotype was highly variable ranging from normal (in the better ear) to profound. These findings align with previous publications [38] and may be due to (i) the *GJB2* variant being coincidental, with HL secondary to variants in another NSHL gene, (ii) failure to detect a second, possibly intronic functionally significant variant in *GJB2*, (iii) the *GJB2* variant modifies the expression of other variants in related HL genes or (iv) the *GJB2* variant being coincidental and the HL stemming from a non-genetic aetiology. Comprehensive panel testing and/or whole genome sequencing may help identify the first two possibilities [43], and further research could possibly elucidate the third.

It is important to appreciate that classification of HL in this study is relative to the better hearing ear. Thus, our study detected individuals with normal hearing and asymmetric hearing loss where the hearing of the contralateral ear could range from mild to profound. The fact that genetic testing was offered in these cases implies that the HL was, at the time of testing, considered to be clinically indicated and/or socially significant to the individual or their families. While HL in some cases may have been complicated by transient, conductive overlay, the findings from the present study support that *GJB*2 variants can be associated with asymmetric HL [8]. The identification of these asymmetric cases (where one ear is classified as ‘normal’ hearing) may be reduced from this point forward given recommendations to only offer genetic testing in cases of bilateral HL [44]. However, a uniform and consistent approach to genetic testing for patients with NSHL is important to mitigate the risk of uncertain findings. Furthermore, this could potentially reduce the financial and psychological costs associated with inappropriate genetic testing.

The natural history in this cohort was predominantly stable but shows both improvements and progression over time. These findings are consistent with those previously reported in the literature [8, 45]. However, it should be noted that audiogram results become more accurate with increasing age in children, thus fluctuation/progression may reflect the young age of this cohort. Importantly, U-shaped HL was identified more frequently at subsequent time points than initial assessments and was associated with c.101 T > C. There is a paucity of literature on U-shaped (mid-frequency) HL, an uncommon audiometric finding, more commonly diagnosed in older individuals [46]. Although U-Shaped HL has not been formally associated with *GJB2* generally and the c.101 T > C variant specifically, in reviewing previously published audiograms in c.101 T > C positive individuals [47], we identified cases of U-Shaped HL. This is clinically significant because this mid-frequency loss is associated with greater difficulty understanding speech in a noisy environment such as a classroom setting. Thus, children may function differentially in quiet and noisy environments, which could mask detection, thus increasing the risk of social problems and fatigue, especially if it is a deterioration [48].

Cumulatively, these results demonstrate a broad phenotypic association with *GJB2* variants and some genotype-phenotype associations which can provide prognostic value. This data from a population wide cohort, provides prognostic information for preconception, prenatal and paediatric counselling of couples and families carrying these variants. For example, Freeman et al.’s [49] discussion of views regarding genetic testing for deafness in reproductive settings, highlighted that the recent American College of Medical Genetics and Genomics practice guidelines [40] recommended the inclusion of *GJB2* variants in prenatal genetic screening on the basis of prevalence and NSHL being categorised as ‘moderately severe’ [50]. If such guidelines were adopted in Australia, the information in studies like this would be invaluable in counselling.

Strengths of this study include the agnostic mode of ascertainment which allowed for identification of a broad phenotypic spectrum. The centralisation of newborn screening, pathology and clinical data allowed for comprehensive phenotypic characterisation. Limitations include a finite sample size, which necessitated the grouping of moderately-severe, severe and profound HL in the analysis. We acknowledge that the impact on quality of life would be different between these groups. Other limitations include a lack of detailed data about other potentially contributing factors for HL and the fact that testing was limited to *GJB2* coding variants and a single *GJB6* deletion.

Future directions to further assist clinicians in providing genetic counselling in this area could include longer follow-up to clarify stability over time, broadening the phenotype to include developmental outcomes including speech and language development and response to intervention e.g., documenting outcomes of children who have required cochlear implants, and comprehensive panel testing for HL. Cumulatively, this information would provide clinicians and families with greater prognostic and management certainty at the time of diagnosis.

## Conclusion

This study provides valuable insights for managing and counselling individuals with *GJB2/GJB6* variants from a population health perspective. The phenotypic spectrum in biallelic individuals in this cohort is milder than has been previously reported, likely due to the agnostic ascertainment. Conversely, our study identified a portion of heterozygous carriers experienced hearing loss, which ranged from mild to moderate. The publication of the full spectrum of presentations offsets the prior publication of more severe presentations, which has potentially skewed the overall perception of the severity of the condition. Given the increasing interest in pre-conception carrier testing for deafness, larger cohort data is crucial to provide personalised, accurate genetic counselling.

## Supplementary information

Supplementary material

## Data Availability

The data are available in Table 1 and Supplementary Table 2 and the variants have been submitted to ClinVar SUB13514054 in July 2023.
