# Supplementary material for: The congenital hearing phenotype in GJB2 in Queensland, Australia: V37I and mild hearing loss predominates
Source: Eur J Hum Genet. 2024 Mar 15;33(2):208–19. doi: 10.1038/s41431-024-01584-0 (PMC11840006; doi:10.1038/s41431-024-01584-0)
Supplement: Supplementary file 1 — Supplementary material [file 41431_2024_1584_MOESM1_ESM.docx]

Supplementary material:

**Supplementary Table 1. Definition of Audiology Descriptors.**

| **Hearing Loss Classification**^†^ **(Goodman and adapted by Clark)** | | **Hearing Threshold in better hearing ear decibels (dB)** | |
| --- | --- | --- | --- |
| Normal | | <20 dB | |
| Mild | | 21-40 dB | |
| Moderate | | 41-55 dB | |
| Moderately-Severe | | 56-70 dB | |
| Severe | | 71-90 dB | |
| Profound | | >90 dB | |
|  |  |  | |
| **Description**^‡^ | | | |
| Rising | | ≥ 15dB HL difference between the average thresholds at 500Hz and the (better) average thresholds at 4 kHz | |
| Sloping/Descending | | ≥15dB HL difference between the (better) average thresholds at 500Hz and those at 4 kHz | |
| Flat | | ≤15db HL difference between all thresholds 0.5- 4kHz | |
| U-shaped | | one or more adjacent thresholds between 500 and 4000 Hz were ≥ 20 dB relative to the better threshold at 250 or 8000 Hz. | |
| Symmetrical | | Both ears are the same | |
| Asymmetrical | | Hearing thresholds differed with a HL difference ≥10dB at a minimum of four frequencies, a HL difference ≥ 15dB at 2 frequencies or a HL difference ≥25 dB at one frequency | |
|  | | |  |
| **Stability** ^‡^ | | | |
| Fluctuating | | Change in hearing threshold of 15dB or greater at any octave frequency between 0.5 and 4kHz but subsequently recover over the period of investigation | |
| Progressive | | A decrease in 10dB or greater at 2 or more adjacent frequencies between 0.5 and 4KHz or a decrease in 15db at one octave frequency in the same frequency range over the period of investigation. | |
| Stable | | Longitudinal audiograms that do not demonstrate the changes specified above | |
| Insufficient information | | Only one audiogram available | |

†Adapted from Goodman and Clark [19, 20]

‡ Adapted from Guo and Pittman [21, 22]

**Supplementary material Figure 1: severity of HL initial and latest**

**a) Severity of HL initial and latest: hmz/comp het/AD n=80**

Hmz=homozygous, comp het = compound heterozygous, AD = autosomal dominant

**b) Severity of HL initial and latest: het/VUS/LB**

N=47

Het=heterozygous, VUS= variant of unknown significance, LB = likely benign

**Supplementary Table 2: Genotypes and phenotypes with age at genetic testing for individuals with compound heterozygous (one pathogenic), and heterozygous variants in *GJB2* NM_4004.5 (NP_003995.2) n=47**

| Zygosity | Age at genetic testing (years) | Variant(s) | Initial HL degree (better ear) | Latest HL degree (better ear) | ACMG | ClinVar | GnoMAD MAF |
| --- | --- | --- | --- | --- | --- | --- | --- |
| Compound heterozygous (1 pathogenic variant) | 1 years, 0 months | c.109G>A p.(Val37Ile) c.571T>C p.(Phe191Leu) | normal | mild | P VUS | P VUS | 0.007 0.0001 |
|  | 0 years, 4 months | c.109G>A p.(Val37Ile) c.265C>T p.(Leu89Phe) | normal | normal | P VUS | P VUS | 0.007 0.00002 |
|  | 0 years, 2 months | c.-45C>A c.79G>A p.(Val27Ile) | mild | missing | P B | P(32), B(1), LB(1)/ B | 0.002 0.05 |
| Heterozygous for Pathogenic/Likely Pathogenic Variants | 0 years, 5 months | c.-45C>A | normal | normal | P | P(32), B(1), LB(1) | 0.002 |
|  | 15 years, 6 months | c.101T>C p.(Met34Thr) | mild | mild | P | P | 0.008 |
|  | 5 years, 11 months |  | mild | mild |  |  |  |
|  | 3 years, 6 months |  | normal | mod-severe |  |  |  |
|  | 0 years, 3 months |  | mild | mild |  |  |  |
|  | 0 years, 5 months |  | mod | mod |  |  |  |
|  | 0 years, 1 months |  | mod | mod-severe |  |  |  |
|  | 2 years, 8 months | c.109G>A p.(Val37Ile) | mild | mild | P | P | 0.007 |
|  | 0 years, 11 months |  | mod | missing |  |  |  |
|  | 0 years, 1 months |  | mild | mild |  |  |  |
|  | 0 years, 5 months |  | mild | mild |  |  |  |
|  | 0 years, 2 months |  | mild | mild |  |  |  |
|  | 0 years, 2 months | c.250G>A p.(Val84Met) | profound | profound | P | P | 0.000004 |
|  | 1 years, 11 months | c.313_326del p.(Lys105Glyfs*5). | normal | mild | P | P | 0.0001 |
|  | 0 years, 5 months | c.35delG  p.(Gly12Valfs*2) | mild | mod | P | P | 0.005 |
|  | 0 years, 3 months |  | normal | missing |  |  |  |
|  | 4 years, 7 months |  | mild | mild |  |  |  |
|  | 0 years, 2 months |  | mild | mild |  |  |  |
|  | 0 years, 4 months |  | normal | normal |  |  |  |
|  | 12 years, 3 months | c.487A>G p.(Met163Val) | mild | mild | P | VUS | 0.0001 |
|  | 0 years, 4 months | c.514T>A p.(Trp172Arg) | normal | normal | LP | P/VUS | 0.00002 |
|  | 0 years, 2 months | c.-22-2A>C | mild | normal | LP | LP | 0.0006 |
| Heterozygous for VUS/Benign/Likely Benign | 0 years, 2 months | c.11G>A p.(Gly4Asp) | mild | mild | VUS |  | 0.00004 |
|  | 1 years, 3 months | c.380G>A p.(Arg127His) | mild | mod | VUS | VUS | 0.001 |
|  | 0 years, 11 months | c.503A>G p.(Lys168Arg) | mild | mild | VUS | VUS(7)/ LB(1) | 0.00005 |
|  | 0 years, 2 months | c.-130C>G | normal | normal | not reported | not found | not found |
|  | 0 years, 3 months | c.50C>G p.(Ser17Cys) | mild | mild | not reported | not found | not found |
|  | 3 years, 7 months | c.-216T>G p.(=) | mod | mod-severe | LB | LB/B | 0.01 |
|  | 0 years, 5 months | c.341A>G  p.(Glu114Gly)  c.79G>A p.(Val27Ile) | normal | missing | B B | LB B | 0.01 0.05 |
|  | 3 years, 3 months | c.79G>A p.(Val27Ile) | normal | severe | B | B | 0.05 |
|  | 2 years, 3 months |  | mild | missing |  |  |  |
|  | 2 years, 2 months |  | mild | mild |  |  |  |
|  | 0 years, 2 months |  | normal | missing |  |  |  |
|  | 0 years, 2 months |  | mod | mod |  |  |  |
|  | 0 years, 4 months |  | normal | normal |  |  |  |
|  | 0 years, 3 months |  | normal | normal |  |  |  |
|  | 0 years, 2 months |  | severe | severe |  |  |  |
|  | 0 years, 3 months |  | normal | normal |  |  |  |
|  | 0 years, 4 months |  | mild | mild |  |  |  |
|  | 0 years, 3 months |  | mild | missing |  |  |  |
|  | 0 years, 2 months | c.608T>C p.(Ile203Thr) | mod | mod | B | B | 0.004 |
|  | 7 years, 0 months | c.249C>Gp.(Phe83Leu) | mod-severe | mod-severe | B | LB | 0.001 |
|  | 0 years, 2 months |  | mod | mod |  |  |  |
|  | 0 years, 2 months |  | mod | mod |  |  |  |
